# Sharp Increase in Eating Disorders among University Students since the COVID-19 Pandemic: Marie-Pierre Tavolacci

**DOI:** 10.1093/eurpub/ckac129.549

**Published:** 2022-10-25

**Authors:** M Tavolacci, J Ladner, P Déchelotte

**Affiliations:** 1 CIC 1404, CHU Rouen, Rouen, France; 2 DEPS, CHU Rouen, Rouen, France; 3 Department of Nutrition, CHU Rouen, Rouen, France; 4 INSERM 1073, Université Rouen, Rouen, France

## Abstract

**Background:**

The COVID-19 pandemic has caused stress, required lockdowns, curfews and social restriction and thus may have altered physical activity and eating habits among university students. The objectives were to determine the impact of the COVID-19 pandemic on Eating Disorders (ED) among university students.

**Methods:**

Between 2009 and 2021, five repeated on line cross-sectional studies were conducted among university students. ED were identified using the French version of the five-item “Sick, Control, One stone, Fat, Food” (SCOFF) questionnaire. The ExpaliTM-validated algorithmic tool, combining SCOFF and body mass index, was used to screen EDs into four diagnostic categories: bulimic, hyperphagic and restrictive ED.

**Results:**

With the five studies, 8981 university students were included in total, 67.3% female with a mean age of 20.7 years. The prevalence of ED was stable between 2009 and 2018 and significantly increased from 31.8% in 2018 to 51.8% in 2021 for women (p trend < 0.0001), and from 13.0% in 2009 to 31.3% in 2021 for men (p trend < 0.0001). Lower food security scores were associated with a higher risk for all ED categories. Depression and academic stress due to COVID-19 were associated with ED regardless of category. Regarding health behaviors, a high adherence to the National nutrition recommendation was a protective factor for the risk of bulimic ED, hyperphagic ED and restrictive ED. A lower frequency of moderate and vigorous physical activity was associated with a higher risk of hyperphagic ED.

**Discussion:**

Our study has shown a high screening of ED among the students of a French university fourteen months after the beginning of the COVID-19 pandemic. By disrupting academic learning, jobs and social life, the COVID-19 pandemic could have exacerbated existing ED or contributed to the onset of new ED. Initiatives to reinforce early screening of ED to implement targeted interventions in the student population are urgently needed.

**Key messages:**

• This finding underlines the need to extensively screen for ED in students population and identify vulnerable individuals at risk of ED.

• By disrupting academic learning, jobs and social life, the COVID-19 pandemic could have exacerbated existing ED or contributed to the onset of new ED.

